# No effect of tattoos on local sweat concentrations of select cytokines, cortisol, glucose, blood urea nitrogen, or lactate during exercise

**DOI:** 10.1038/s41598-024-63057-0

**Published:** 2024-05-31

**Authors:** James R. Merritt, Michal Ozga, Peter John D. De Chavez, Ali Boolani, Lindsay B. Baker

**Affiliations:** 1 Gatorade Sports Science Institute, PepsiCo R&D, Valhalla, NY USA; 2 Data Science & Analytics, PepsiCo R&D, Plano, TX USA

**Keywords:** Biomarkers, Biochemistry

## Abstract

Due to growing interest in the investigation of exercise induced sweat biomarkers to assess an individual’s health and the increasing prevalence of tattoos in the world’s population, investigators sought to determine whether local sweat concentrations and excretion rates of epidermal growth factor (EGF), interleukin (IL) -1α, IL-6, IL-8, cortisol, glucose, blood urea nitrogen (BUN), and lactate differ between tattooed and contralateral non-tattooed skin during exercise. Sixteen recreational exercisers [female (50%)] (age = 25–48 years) with ≥ 1 unilateral permanent tattoo [median tattoo age = 6 years, IQR = 5] on the arm/torso completed an outdoor group fitness session. There were no significant differences between tattooed and non-tattooed skin for sweat EGF, IL-1α, IL-8, cortisol, glucose, BUN, or lactate concentrations. There were no significant differences between tattooed and non-tattooed skin for sweat EGF, IL-1α, IL-8, cortisol, glucose, BUN, or lactate excretion rate. Findings suggest that permanent tattoos older than 1 year may not impact local sweat EGF, IL-1α, IL-8, cortisol, glucose, BUN, and lactate concentrations or excretion rates during exercise.

Clinical trial identifier NCT04920266 was registered on June 9, 2021.

## Introduction

Human sweat contains a wealth of biomarkers that may offer insights into physiological functions and athletic performance. These biomarkers include electrolytes, such as sodium and chloride; interleukins (IL); and metabolites, such as glucose, lactate, cortisol, ammonia, and blood urea nitrogen (BUN), which can be non-invasively measured from a sweat sample^1^. Specifically, cytokines in sweat may have the potential to serve as immune function markers^2–5^ and indicators of local skin inflammation^6,7^. Meanwhile, as heightened levels can suppress immune function and promote various maladaptations^8^, monitoring cortisol may aid in understanding an individual’s recovery response to various stressors (i.e., strenuous exercise training, illness, and disease). Therefore, the introduction of skin interfaced platforms capable of analyzing sweat chemistry has ushered in a shift from traditional blood biomarker testing to sweat analyses^9^.

As the scientific community delves into this avenue of research, it is essential to account for potential confounders that may impact sweat composition, including the impact of tattoos. Tattoos are increasingly common among physically active individuals, including athletes^10,11^, military personnel^12,13^, and tradespeople^14^; and it is important to understand the effect that the tattooing process has on sweat and sweat gland function. Although the prevalence of tattoos in the general population has been reported to be relatively low (~ 10–20%)^14,15^, up to 53% of players in the National Basketball Association (NBA) (NBATattoos, 2016), 33% of players at the 2018 Fédération Internationale de Football Association (FIFA) World Cup^11^, and 36% of military recruits^12^ have at least one permanent tattoo. One recent study reported that the prevalence of tattoos for the general population in the United States is rising, with 32% of Americans having at least one tattoo, and the highest rates being 46% and 41% for those between the ages of 30–49 years and 18–29 years, respectively^16^.

The tattooing process involves injecting ink into the skin’s dermal layer through repeated microneedle punctures potentially affecting eccrine sweat glands, which are primarily located in the dermis. This interaction may compromise gland function, leading to changes in sweat biomarker concentrations and excretion rates. Previous studies have suggested that the tattooing process results in an inflammatory response and elevated cytokine release in the tattooed area^17–19^. While one study reported increased IL-18, IL-1α, and IL-8 release after 24-h exposure to various tattoo inks^18^, another study reported that chronic release of inflammatory cytokines is unlikely^19^. However, to the authors’ knowledge the persistence of inflammatory cytokine release in healed tattooed skin (> 6 months) remains largely unexplored (Millhone, 2023).

Existing literature focused on the potential effects of tattoos on sweat biomarkers is limited to sweating rate and sweat electrolyte concentrations, with several studies reporting no differences between tattooed skin (TAT) and non-tattooed skin (NT) during exercise^20–22^. To the authors’ knowledge, studies investigating the effects of tattoos on local sweat concentrations and excretion rates of inflammatory cytokines, cortisol, glucose, BUN, and lactate, are lacking. Therefore, research investigating the concentrations of these analytes within sweat of TAT compared to contralateral NT areas is warranted. Moreover, given the recent advances in the use of wearable biosensors for sweat biomarker analysis^23^, it is imperative to discern the impact of external confounders such as tattoos on sweat composition. Therefore, the purpose of this exploratory study was to determine if local sweat concentrations and excretion rates of epidermal growth factor (EGF), IL-1α, IL-6, IL-8, cortisol, glucose, BUN, and lactate, differ between TAT and contralateral NT areas during a group fitness exercise session.

## Methods

### Subjects

Seventeen healthy recreational exercisers [female (53%)] with at least one permanent, unilateral tattoo on the arm or torso voluntarily enrolled in this study. Subjects were recruited from the Chicago, IL metropolitan area and were moderately trained (i.e., engaged in regular outdoor fitness (~ 1 h) sessions at least twice a week). Participants were excluded if they met any of the following criteria: (1) were pregnant; (2) smoked; (3) had an allergy to adhesives; or (4) had ≥ two cardiovascular risk factors. Due to limited sweat sample volume, six subjects were excluded (one entirely and five partially) from the final analysis. This impacted the final sample size for all cytokines (n = 16), cortisol (n = 13), glucose (n = 13), BUN (n = 12), and lactate (n = 11). Participants were informed of all experimental procedures and associated risks before providing written informed consent. This study (clinical trial identifier: NCT04920266) was approved by the Sterling Institutional Review Board (Atlanta, GA, USA; sterlingirb.com) and was completed in accordance with the ethical standards in the Declaration of Helsinki.

### Experimental design

In this cross-sectional study, data were collected during two 60-min outdoor, instructor-led group fitness sessions in August and October in Chicago Illinois, USA. Each participant completed one experimental trial during which the contralateral side of the TAT area served as the control. This was considered a fair comparison since previous studies have reported no significant bilateral differences in local sweating rate^24–26^ or sweat electrolyte concentrations^27,28^.

Sweat samples analyzed in this study were collected using the protocol described in Keyes et al. While the electrolyte data from these sweat samples were published previously^20^, the cytokine, lactate, cortisol, BUN, and glucose data have not been published elsewhere. The National Institutes of Health’s quality assessment tool for observational cohort and cross-sectional studies was employed to assess the study’s internal validity (see Supplementary Table S1 online).

Median ± Interquartile Range (IQR) tattoo age was 6 ± 5yrs and the ink shading density was 59 ± 27%. Tattoos were either black (n = 10) or multicolored (n = 6) and were located on the scapula (n = 4), triceps (n = 3), forearms (n = 3), wrists (n = 2), biceps (n = 2), shoulder (n = 1), and torso (n = 1). During the two outdoor sessions, air temperature (27 ± 3  °C), relative humidity (RH; 63 ± 4%), and wet bulb globe temperature (WBGT; 26 ± 3 °C) were measured using a Kestrel 5400 (Nielsen-Kellerman Co., Boothwyn, PA, USA). Heat acclimation status was not assessed, but since subjects were tested in the summer and autumn months, they may have been partially to fully heat acclimated. Due to the exploratory nature of this study, a power calculation was not completed.

Prior to testing, participants body mass was measured to the nearest 0.05kg (BC-350; Tanita Corporation, Arlington Heights, IL, USA) while wearing minimal clothing (i.e., men in compression shorts and women in compression shorts and a sports bra). Each participant was equipped with a global positioning system device (Garmin Forerunner® 245, Garmin International, Inc., Olathe, KS, USA) to collect heart rate, energy expenditure, and exercise duration. Energy expenditure was calculated via a proprietary algorithm developed by Garmin and Firstbeat Analytics using heart rate, respiration rate derived from heart rate variability, and another non-disclosed variable. Participants could eat and drink ad libitum during the exercise session. All food products and drink bottles were weighed before and after consumption to determine amounts consumed to the nearest 1g (CS2000, Ohaus, Pine Brook, NJ, USA). Following the exercise, participants were asked to towel dry before being weighed again, with the same scale and manner (minimal clothing) as the pre-exercise measurement.

### Local sweat collection

Prior to exercise, the sweat patch sites were shaved as needed, cleaned with alcohol, and air dried. Absorbent patches (Tegaderm™ + Pad, 3M, St. Paul, MN, USA) were then applied to the TAT and NT areas. Contralateral patch placement sites were estimated visually and via direct side-by-side comparison when possible (i.e., by bringing the left and right triceps, forearms, and wrists together). To enhance adhesion, Surgilast™ (Derma Sciences, Princeton, NJ, USA) was used when applicable, particularly on the forearms or wrists.

Throughout the exercise, investigators regularly inspected the patches visually for adhesion and sample volume. To maintain quality control, the patches’ adherence to the skin was visually assessed immediately before and during the removal process, ensuring there was no evaporation of the sample due to separation between the Tegaderm™ and skin during the exercise session. When patches became moderately saturated, they were removed from both the TAT and NT areas. Absorbent pads were immediately removed from the Tegaderm™ using clean forceps and placed in an air-tight plastic tube (Sarstedt Salivette, Nümbrecht, Germany). The tubes were sealed with Parafilm™ (Bemis Company, Inc., Neenah, WI, USA), placed in a plastic bag, and transported to the laboratory for subsequent processing and analysis. In the field and during transportation, samples were stored in an ice chest (~ 10 °C) and subsequently transferred to a laboratory refrigerator (2–3 °C). When overnight storage was needed, sample analysis was completed within 48 h.

### Multiplex assay

EGF, IL-1α, IL-1β, IL-6, and IL-8 in the athletes’ sweat were measured using magnetic bead-based immunoassay Multiplex. The Multiplex assay was performed in accordance with the manufacturer’s instructions (Milli-Q, Millipore Sigma, Burlington, MA, USA). Sweat was extracted from pads via centrifuge, and 25µL of each sweat sample was assayed in duplicate. The Multiplex assay involved adding protein-specific antibodies affixed to distinctly colored beads to the sweat samples in the wells of a microplate. These target proteins were left to bind with the capture antibodies during an overnight incubation. Subsequently, the beads were manually washed with a magnetic plate, and underwent a 1-h incubation period with protein-specific biotinylated detector antibodies. Immediately after incubation, excess biotinylated detector antibodies were removed. R-phycoerythrin (SAV-RPE), a streptavidin-conjugated fluorescent protein, was added, and an additional 30-min incubation period allowed the SAV-RPE to form a four-member solid-phase sandwich with the biotinylated detector antibodies. After the final incubation period, the beads were washed again to remove any unbound SAV-RPE. The beads were then analyzed using the Magpix multiplex system with xPonent software (Luminex, Austin, TX, USA) to determine individual analyte concentrations (pg/mL).

### ELISA assays

ELISA was used for the detection of cortisol (Invitrogen, ThermoFisher Scientific, Waltham, MA, USA), glucose (Cayman Chemical Co., Ann Arbor, MI, USA), BUN (Invitrogen, ThermoFisher Scientific, Waltham, MA, USA), and lactate (LSBio, Seattle, WA, USA). All ELISA assays were performed in accordance with the manufacturers’ instructions.

Cortisol, glucose, BUN, and lactate were all assayed in duplicate. For cortisol analysis, 12.5 µL of each sweat sample was assayed to detect non-specific binding. The assay buffer, cortisol conjugate, and cortisol antibody were added to the samples in each well and incubated for 1-h at room temperature (~ 20 °C). After four manual washes, tetramethylbenzidine substrate was added and incubated for 30-min, producing a colorimetric signal. Stop solution was added for 10-min and the absorbance was read at 450 nm using a microplate reader. For glucose measurement, 15 µL of each sweat sample was mixed with the assay buffer in each well. The reaction was initiated by adding the glucose enzyme mixture, and the microplate was covered and incubated for 10-min at 37 °C. Absorbance was read at 520 nm using a microplate reader. For BUN analysis, 15 µL of each sweat sample was assayed. Two color reagents were added to each well, and the microplate was covered and incubated at room temperature (~ 20 °C) for 30-min. Absorbance readings were taken at 450nm using a microplate reader. For lactate measurement, 12.5 µL of each sweat sample was combined with the assay buffer in each well. The reaction was initiated by adding the lactate enzyme mixture, and the microplate was covered and incubated for 30-min at 37 °C. Absorbance was read at 590 nm using a microplate reader. All assays were read using Biotek Cytation 3 Multi-Reader (Biotek, Winooski, VT, USA) with Gen5 software.

### Calculations

Local sweating rate (LSR; mg/cm^2^/min) was calculated by quantifying the sweat mass absorbed in each pad over the pad surface area (11.9 cm^2^) and duration on skin. Sweat mass absorbed in the pad was calculated by taking the difference between the initial patch mass before application from the mass after removal to the nearest 0.001 g. This measurement was performed using an analytical balance (Mettler Toledo Balance XS204, Columbus, OH, USA). Local excretion rates (pg/cm^2^/min or ng/cm^2^/min) were calculated by multiplying the local analyte concentration by the corresponding LSR. Whole body sweating rate (WBSR; L/h) was calculated from the difference in pre to post exercise body mass, adjusted for estimated respiratory water loss, estimated metabolic mass loss^29^, and fluid intake (difference in drink bottle mass from pre to post exercise to the nearest 0.001kg) using a digital scale; model PG802 Mettler Toledo, Columbus. OH, USA).

### Statistical analysis

Statistical analyses were conducted using Minitab 19 Statistical Software (Minitab, Inc., State College, PA, USA). Data are presented as mean ± standard deviation (SD) or median ± IQR for non-normally distributed data. To evaluate differences between TAT and NT skin, paired *T*-tests or Wilcoxon Signed-Rank tests were used, and normality was assessed using the Shapiro–Wilk’s test. For all statistical tests, the significance level was set at α = 0.05. Coefficient of variation values for biomarker concentrations are included in Supplementary Table S2.

## Results

### Subject characteristics

Participants’ (n = 16, male = 8, female = 8) average age, pre-exercise body mass, heart rate and energy expenditure during the exercise bout, and body mass lost pre- vs. post-exercise were 33 ± 8 years, 77.60 ± 11.00 kg, 153 ± 16 bpm, 707 ± 155 kcal, and 1.39 ± 0.31%, respectively. The mean duration of exercise was 56 ± 2 min.

### Sweating rate

**Figure 1:**
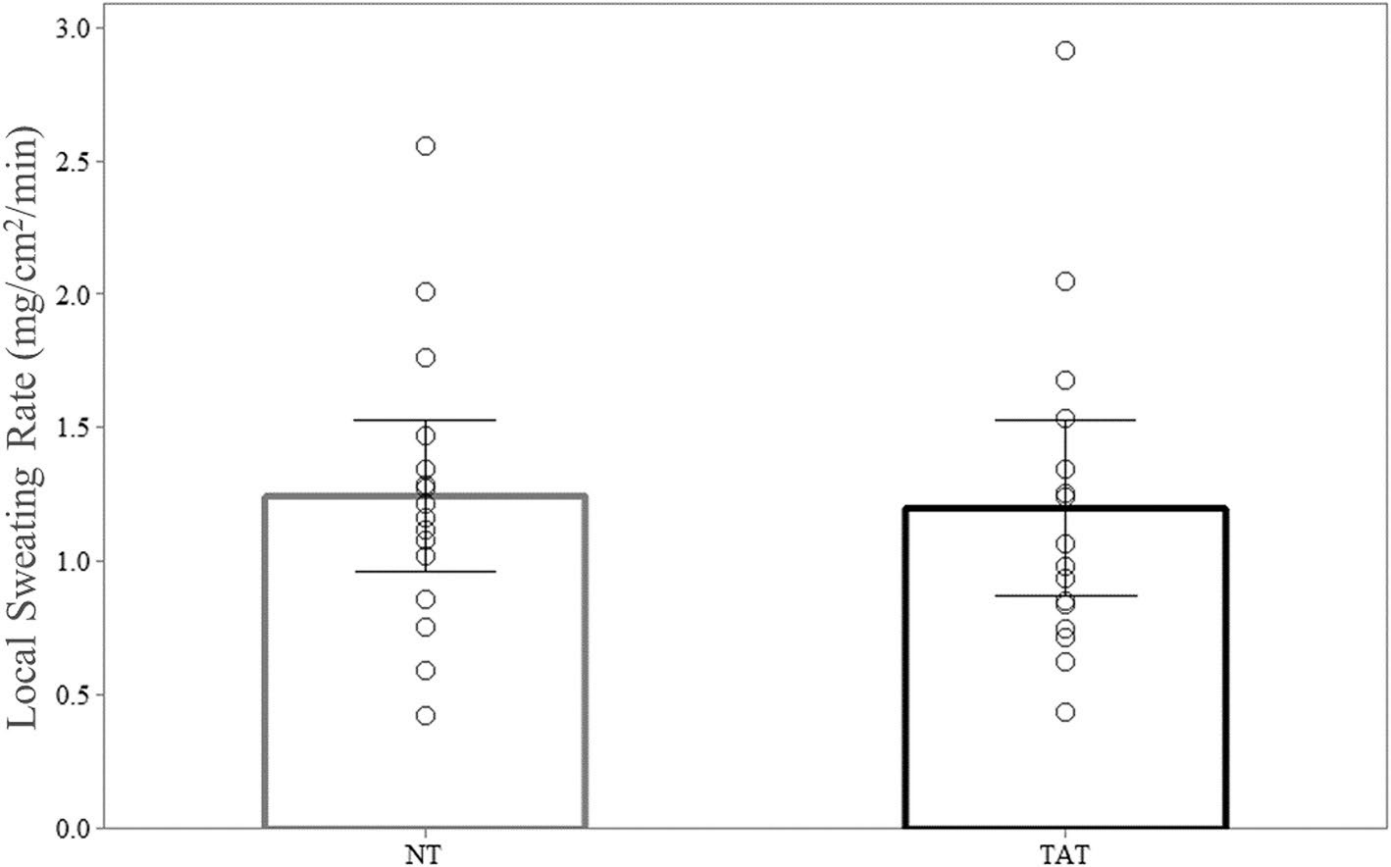
No differences in local sweating rate between tattooed (TAT) and non-tattooed (NT) sites (Mean ± SD). Paired T-Test (*P* > 0.05).

Mean WBSR during exercise was 1.01 ± 0.31L/h. Mean LSR (n = 16) for TAT (1.20 ± 0.62mg/cm^2^/min) was not significantly different (*P* = 0.41) than NT (1.25 ± 0.53mg/cm^2^/min) (Fig. 1).

### Sweat cytokines

**Figure 2:**
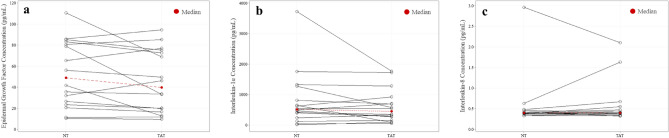
No differences in local sweat cytokine concentrations between tattooed (TAT) and non-tattooed (NT) sites. Wilcoxon signed-rank test for (**a**) epidermal growth factor, (**b**) interleukin (IL)-1α, and (**c**) IL-8 (Median). (*P* > 0.05 for all).

**Figure 3:**
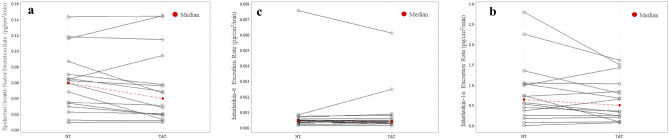
No differences in local sweat cytokine excretion rates between tattooed (TAT) and non-tattooed (NT) sites. Wilcoxon signed-rank test for (**a**) epidermal growth factor, (**b**) interleukin (IL) -1α, and (**c**) IL-8 (Median). (*P* > 0.05 for all).

Median local sweat EGF (TAT vs NT; 39.80 ± 54.22 pg/mL vs 48.92 ± 55.41 pg/mL), IL-1α (453.60 ± 511.00 pg/mL vs 518.63 ± 565.50 pg/mL), and IL-8 (0.40 ± 0.11 pg/mL vs 0.40 ± 0.06 pg/mL) concentrations were not significantly different (*P* = 0.17–0.29) between TAT and NT (n = 16) (Fig. 2). Median local sweat EGF (TAT vs NT; 0.05 ± 0.05 pg/cm^2^/min vs 0.06 ± 0.04 pg/cm^2^/min), IL-1α (0.64 ± 0.52 pg/cm^2^/min vs 0.84 ± 0.77 pg/cm^2^/min), and IL-8 (0.0004 ± 0.0004 pg/cm^2^/min vs 0.0005 ± 0.0003 pg/cm^2^/min) excretion rates were not significantly different (*P* = 0.09–0.82) between TAT and NT (n = 16) (Fig. 3). IL-6 data were not included since the results fell outside the predetermined limits of sensitivity (0.04pg/mL).

### Sweat cortisol

**Figure 4:**
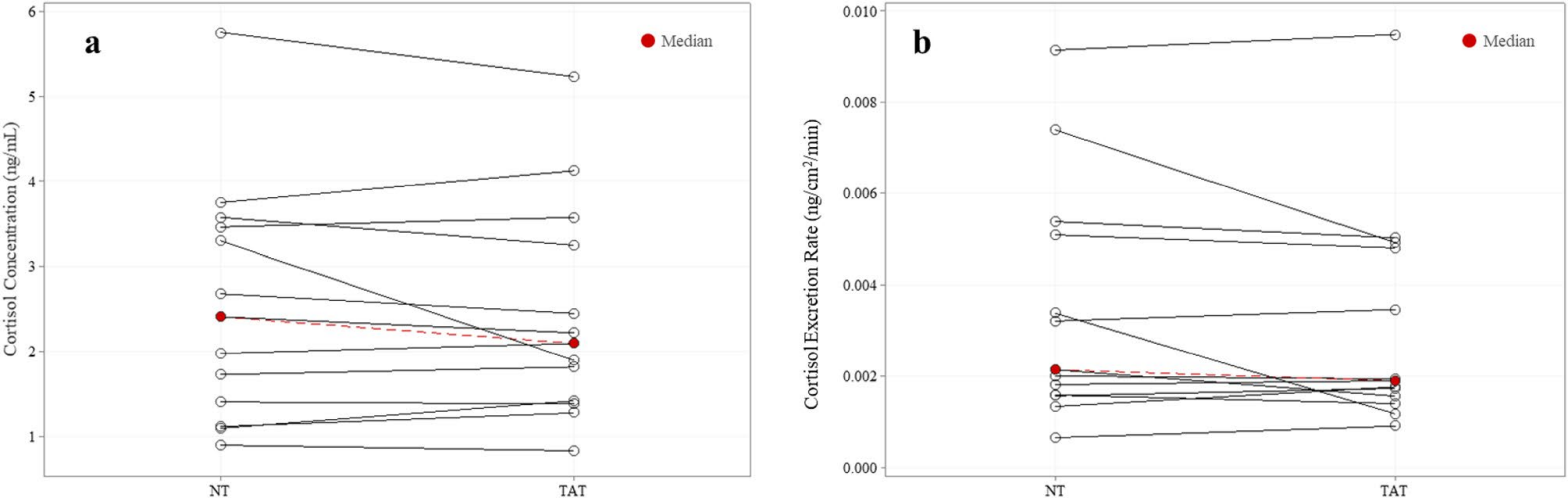
No differences in local sweat cortisol (n = 13) (**a**) concentration or (**b**) excretion rate between tattooed (TAT) and non-tattooed (NT) sites (Median). Wilcoxon signed-rank test (*P* > 0.05 for both).

Median local sweat cortisol concentration (2.10 ± 2.00 ng/mL vs 2.41 ± 2.25 ng/mL) and excretion rates (0.002 ± 0.003 ng/cm^2^/min vs 0.002 ± 0.003 ng/cm^2^/min) were not significantly different (*P* = 0.62, *P* = 0.44) between TAT and NT (n = 13) (Fig. 4).

### Sweat glucose

**Figure 5:**
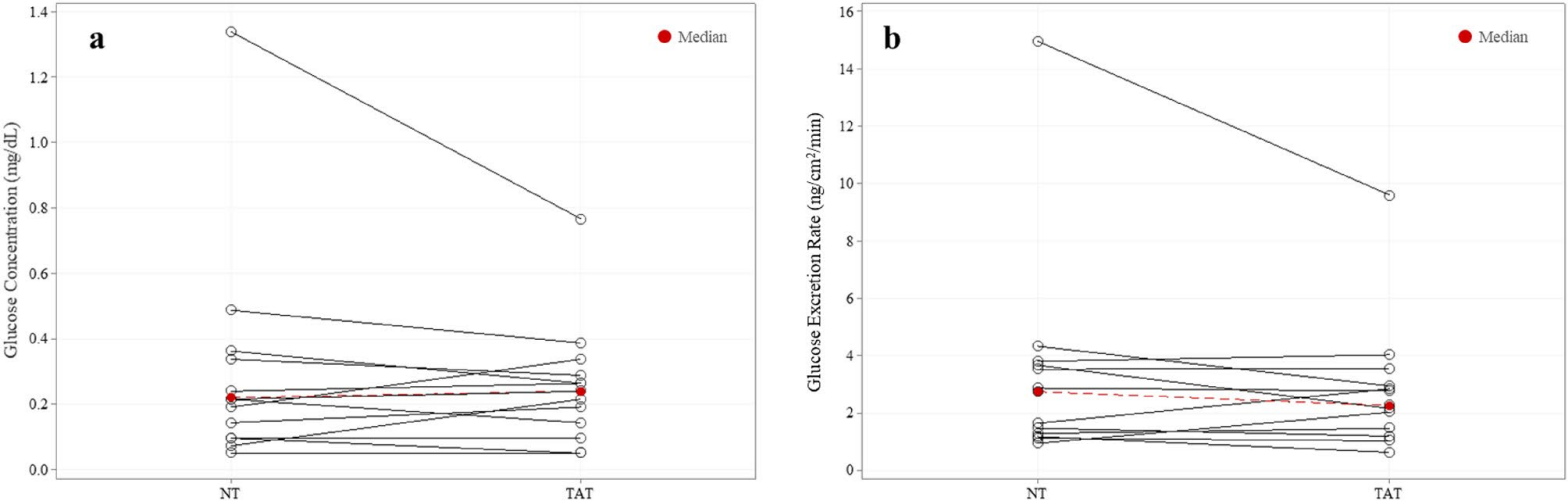
No differences in local sweat glucose (n = 13) (**a**) concentration or (**b**) excretion rate between tattooed (TAT) and non-tattooed (NT) sites (Median). Wilcoxon signed-rank test (*P* > 0.05 for both).

Median local sweat glucose concentration (n = 13) for TAT and NT groups (0.24 ± 0.19 mg/dL vs 0.22 ± 0.25 mg/dL) and excretion rates (2.26 ± 1.92 ng/cm^2^/min vs 2.74 ± 2.50 ng/cm^2^/min) were not significantly different (*P* = 0.56, *P* = 0.26) (Fig. 5).

### Sweat urea nitrogen

**Figure 6:**
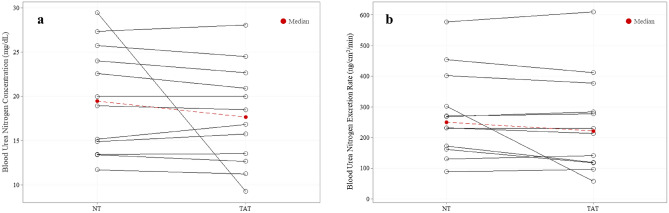
No differences in local sweat blood urea nitrogen (n = 12) (**a**) concentration or (**b**) excretion rate between tattooed (TAT) and non-tattooed (NT) sites (Median). Wilcoxon signed-rank test (*P* > 0.05 for both).

Median local sweat BUN concentration (17.65 ± 9.35 mg/dL vs 19.46 ± 11.49 mg/dL) and excretion rates (221.64 ± 235.80 ng/cm^2^/min vs. 250.05 ± 212.87 ng/cm^2^/min) between TAT and NT (n = 12) areas were not significantly different (*P* = 0.27, *P* = 0.20) (Fig. 6).

### Sweat lactate

**Figure 7:**
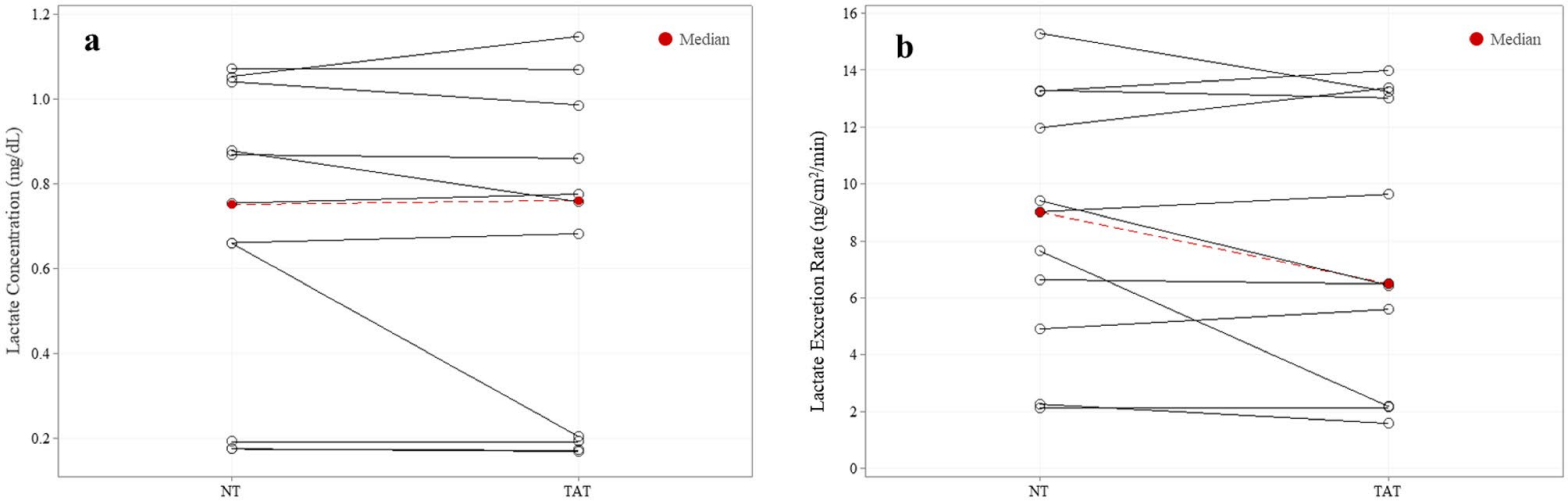
No differences in local sweat lactate (n = 11) (**a**) concentration or (**b**) excretion rate between tattooed (TAT) and non-tattooed (NT) sites (Median). Wilcoxon signed-rank test (*P* > 0.05 for both).

Median local sweat lactate concentration (0.76 ± 0.79 mg/dL vs 0.75 ± 0.85 mg/dL) and excretion rates (6.48 ± 11.07 ng/cm^2^/min vs. 9.01 ± 8.36 ng/cm^2^/min) between TAT and NT (n = 11) areas were not significantly different (*P* = 0.41, *P* = 0.56) (Fig. 7).

## Discussion

Given the growing interest in exercise induced sweat biomarkers to assess health^9^, and the increasing prevalence of tattoos^10–14^, this study investigated whether tattoos affect local sweat analyte concentrations and excretion rates. Our findings suggest that there are no significant differences in LSR, or local sweat concentrations and excretion rates for EGF, IL-1α, IL-8, cortisol, glucose, BUN, or lactate in tattooed skin compared to non-tattooed skin after a single bout of exercise. This study adds to the literature by providing evidence that tattoos may not affect local sweat analyte concentrations and excretion rates during exercise, similar to previous findings for local sweat electrolytes^20–22^.

Wearable sensors have received significant attention in recent years due to the increased demand for personal, real-time health monitoring while technology has continually improved^30^. As popularity for sensor based sweat analyses increases, it is important to understand whether factors such as tattoos may influence the various biomarkers assessed by these technologies. Our findings from the present study suggest that tattoos older than one year may not influence the measures of sweat cytokine and metabolite concentrations. These results are in line with previous work from our group that showed tattoos do not influence LSR and the concentration of potassium in sweat samples, while older tattoos do not impact the concentration of sweat sodium and chloride^20^. Collectively, these findings suggest tattoos older than one year do not impact the rate of regional sweat loss or its constituents.

As many of the constituents of blood are found in sweat^1^, the use of sweat as a non-invasive measure of blood biomarkers may have applications in monitoring health status. However, previous studies have suggested sweat composition may vary from blood composition^1^. For example, following acute submaximal exercise, Klous and colleagues have shown that changes in biomarkers observed in sweat samples were independent of the changes observed in blood^31^. Both physiological (e.g., eccrine gland production, contact with keratinocytes) and methodological (e.g., skin contamination) factors may influence the composition of constituents in sweat, leading to a dissociation between sweat and blood biomarker concentrations. While the possibility of sweat being an accurate surrogate of blood seems low, non-invasive biosensor based sweat analyses may still provide some insights into human physiology, health, and performance^32,33^. Specifically, cytokines in sweat may have the potential to serve as immune function markers^2–5^ and indicators of local skin inflammation^6,7^. With an increase in devices that detect various parameters in sweat, the implications of sweat composition as an indicator of overall health status should continue to be explored in future studies.

The local sweat cortisol, glucose, BUN, and lactate concentrations reported in this study fall within previously reported normative eccrine sweat concentration values^1^. Additionally, previous studies have reported similar local sweat lactate concentrations^34,35^ and local sweat cytokine concentrations and excretion rates for EGF and IL-8^36^ as our study. Further, local sweat cortisol concentration reported in our study is in line with previously reported values following exercise^36,37^ and passive heating^38^. There were differences in local sweat concentrations in the present study versus previous studies for IL-8^2,5^, IL-1α^3^, and BUN^39^. However, methodological differences between those studies and our study may explain the differing concentrations. For example, variations in demographic characteristics (i.e., sex, age, or fitness level) and time of sweat patch adherence^40,41^ may impact sweating rates, which may explain the differing concentrations. Similar effects have been reported between passive and exercise sweat collection^39,42^ with varying exercise intensities (i.e., moderate vs vigorous)^43^ also impacting sweating rates and concentrations. Lastly, both sweat collection methodology (i.e., sweat patches, polyethylene film, or scraping)^1^ and different cytokine analytical techniques (i.e., enzyme immunoassay, multiplex, or recycling immunoaffinity chromatography) have the potential to affect local sweat concentrations. Overall, studies that used similar methodologies reported similar analyte concentrations as our study suggesting that our data are representative of exercise-induced local sweat concentrations of various cytokines and metabolites.

The study has some limitations, including a small sample size, a cross-sectional design, and a heterogenous tattoo sample. Moreover, the direct comparison between the TAT and contralateral NT area was considered a fair comparison since previous studies have reported no significant bilateral differences in local sweating rate^24–26^ or sweat electrolyte concentrations^27,28^. However, future research is needed to determine bilateral variability specifically for sweat cytokine, cortisol, glucose, BUN, and lactate concentrations. Additionally, this study only assessed exercise-induced sweating, therefore future research is needed to determine the effects of tattoos on sweat analyte concentrations using other methods of sweat induction, such as passive heating^44^ or pilocarpine iontophoresis^45^. Finally, since cytokine release is increased during the tattooing process^17–19^, future studies should investigate local sweat analyte concentration differences in newer tattoos (< 1 year).

## Summary

To our knowledge, this was the first study to investigate the effects of tattoos on local sweat cytokine, cortisol, glucose, BUN, and lactate concentrations and excretion rates. Our findings suggest that tattoos may not affect local sweat concentrations and excretion rates of EGF, IL-1α, IL-8, cortisol, glucose, BUN, and lactate during exercise. These results suggest that local exercise-induced sweat sampling and analyses from tattooed skin may be used to interpret cytokine and metabolite data without bias.

### Supplementary Information

Supplementary Tables.

## Data Availability

All data generated or analyzed during this study are included in this article.

## Abbreviations

BUN: Blood urea nitrogen
EGF: Epidermal growth factor
FIFA: Fédération Internationale de Football Association
IL: Interleukin
IQR: Interquartile range
LSR: Local sweating rate
NBA: National Basketball Association
NT: Non-tattooed skin
RH: Relative humidity
SAV-RPE: R-phycoerythrin
SD: Standard deviation
TAT: Tattooed skin
WBGT: Wet bulb globe temperature
WBSR: Whole body sweating rate
