# Supplementary material for: No effect of tattoos on local sweat concentrations of select cytokines, cortisol, glucose, blood urea nitrogen, or lactate during exercise
Source: Sci Rep. 2024 May 31;14:12570. doi: 10.1038/s41598-024-63057-0 (PMC11143332; doi:10.1038/s41598-024-63057-0)
Supplement: Supplementary file 1 — Supplementary Tables. [file 41598_2024_63057_MOESM1_ESM.pdf]

No Effect of Tattoos on Local Sweat Concentrations of Select Cytokines, Cortisol, Glucose, Blood Urea Nitrogen, or Lactate During Exercise

James R. Merritt[1], Michal Ozga[1], Peter John D. De Chavez[2], Ali Boolani[1], Lindsay B. Baker[1]
[1]Gatorade Sports Science Institute, PepsiCo R&D, Valhalla, NY, USA; [2]Data Science & Analytics, PepsiCo R&D, Plano, TX, USA

Supplementary Table S1 Quality Assessment Tool for Observational Cohort and Cross-Sectional Studies

Criteria	Yes<br>Location	No<br>Comments	Other (CD[c], NR[c], NA[c])<br>Comments
1. Was the research question or objective in this paper clearly stated?	☑<br>Introduction		
2. Was the study population clearly specified and defined?	☑<br>Subjects		
3. Was the participation rate of eligible persons at least 50%?	☑<br>Results		
4. Were all the subjects selected or recruited from the same or similar populations (including the same time period)? Were inclusion and exclusion criteria for being in the study prespecified and applied uniformly to all participants?	☑<br>Subjects		
5. Was the sample size justification, power description, or variance and effect estimates provided?		☒<br>Exploratory	
6. For the analysis in this paper, were the exposure(s) of interest measured prior to the outcome(s) being measured?		☒<br>Cross-sectional	
7. Was the timeframe sufficient so that one could reasonably expect to see an association between exposure and outcome if it existed?	☑<br>Experimental Design		
8. For exposures that can vary in amount or level, did the study examine different levels of the exposure as related to the outcome (e.g., categories of exposure, or exposure measured as continuous variable)?	☑<br>Experimental Design		
9. Were the exposure measures (independent variables) clearly defined, valid, reliable, and implemented consistently across all study participants?	☑<br>Experimental Design		

10. Was the exposure(s) assessed more than once over time?			NA Cross-sectional
11. Were the outcome measures (dependent variables) clearly defined, valid, reliable, and implemented consistently across all study participants?	☑ Results		
12. Were the outcome assessors blinded to the exposure status of participants?	☑ Experimental Design		
13. Was loss to follow-up after baseline 20% or less?			NA Cross-sectional
14. Were key potential confounding variables measured and adjusted statistically for their impact on the relationship between exposure(s) and outcome(s)?		☒ Exploratory	

Note. In order to make this table more user-friendly, we added the Location for when we satisfied a criterion and the Comments for when we did not; [c] CD = cannot determine; NA = not applicable; NR = not reported

Supplementary Table S2: Reproducibility of assessing biomarker concentrations in sweat

	**Average** ***(mean ± standard deviation)***	**Range**	***Coefficient of Variance***
**Epidermal growth factor**	**48.86 ± 28.99 pg/mL**	**9.57 – 110.50 pg/mL**	**7.18%**
**IL-1α**	**712.89 ± 736.36 pg/mL**	**15.48 – 3720.00 pg/mL**	**8.43%**
**IL-8**	**0.58 ± 0.56 pg/mL**	**0.32 – 2.96 pg/mL**	**7.33%**
**Cortisol**	**2.49 ± 1.28 ng/mL**	**0.83 – 5.75 ng/mL**	**2.53%**
**Glucose**	**0.27 ± 0.26 mg/mL**	**0.05 – 1.34 mg/mL**	**1.51%**
**Urea nitrogen**	**18.77 ± 5.70 mg/dL**	**9.25 – 29.46 mg/dL**	**0.63%**
**Lactate**	**0.66 ± 0.35 mg/dL**	**0.17 – 1.15 mg/dL**	**1.73%**
